# DMD/BMD prenatal diagnosis and treatment expectation in a single centre in China for 15 years

**DOI:** 10.1186/s12920-021-01024-8

**Published:** 2021-07-08

**Authors:** Xingjian Zhong, Siying Cui, Lina Liu, Yuxia Yang, Xiangdong Kong

**Affiliations:** The Genetics and Prenatal Diagnosis Center, The First Affiliated Hospital of Zhengzhou University, No. 1, Jianshe East Rd., Erqi District, Zhengzhou, Henan Province China

**Keywords:** Duchenne muscular dystrophy (DMD), Prenatal diagnosis, Multiplex ligation-dependent probe amplification (MLPA), Next-generation sequencing (NGS), Sanger sequencing, Short tandem repeat (STR), Gene therapy

## Abstract

**Objective:**

DMD/BMD prenatal diagnosis for 931 foetuses.

**Background:**

DMD is the most common fatal X-linked recessive muscular disease. There is no effective clinical treatment method at present. Accurate gene diagnosis and prenatal diagnosis technology are important ways for early detection, early prevention and early treatment.

**Methods:**

A total of 931 prenatal diagnoses were performed for pregnant women with a definite family history of DMD or a history of DMD childbirth between 2005 and 2019. This report may be considered the largest *DMD* prenatal diagnosis report in a single centre worldwide. Multiple ligation-dependent probe amplification (MLPA) and next-generation sequencing were used in combination. Techniques and short tandem repeat (STR) linkage analysis were used to determine the location of the DMD gene mutation in the pregnant woman and then to detect the *DMD* gene in the foetuses.

**Results:**

There were 872 families in our study. Among all 931 foetuses, 20.73% (193/931) were males expected to develop DMD and 16.33% (152/931) were female carriers. In addition, gonadal mosaicism was observed in 5 mothers, and gene recombination was identified in three foetuses. The results of the prenatal diagnosis were consistent with the results of the CPK analysis, and the results of the prenatal diagnosis were 100% accurate.

**Conclusions:**

MLPA and Sanger sequencing, when combined with STR linkage analyses, can provide an accurate and rapid prenatal diagnosis. Due to the high de novo rate, prenatal diagnosis and genetic counselling should be given great attention.

## Background

Duchenne muscular dystrophy (DMD, OMIM: 310200), the most common X-linked recessive inherited muscle disease, affects approximately 0.02% of all newborn males worldwide [1–3]. In males affected with DMD, symptoms such as progressive muscle weakness manifest around the age of 4–7 and ultimately result in the inability to walk around the age of 12. The age at diagnosis is approximately 5 years when early symptoms occur [4]. Affected children approximately 12 years of age with progressive muscle weakness rely on wheelchairs, and they often eventually prematurely die of respiratory or cardiac failure in the second decade. Becker Muscular Dystrophy (BMD, OMIM: 300376) is milder, with later symptom occurrence, slower disease progression, and fewer effects on survival than DMD; however, it leads to poor quality of life. These phenotypes of BMD usually do not affect the lifespans of patients but seriously affect their quality of life. A precise and accurate prenatal diagnosis is currently the only effective way of preventing this disease.

DMD is caused by structural and functional changes in dystrophin induced by mutations of the DMD gene (OMIM: 300377), which is located on Xp21.1 and represents the largest gene known in humans. The DMD gene spans approximately 2.4 Mb of genomic DNA and contains 79 exons and 78 introns [5], generating a 14 kb mRNA transcript and producing a 527 kDa dystrophin protein, which is a cytoskeletal protein that enhances muscle fibre strength, stability and function [6]. Above all, its high mutation rate might be related to its length [5]. In our study, multiplex ligation-dependent probe amplification (MLPA), next-generation sequencing (NGS) and Sanger sequencing, combined with short tandem repeat (STR) linkage analyses, were used for prenatal diagnosis.

## Subjects and methods

### Subjects

Between January 1, 2005, and December 31, 2019, 931 foetuses from 927 pregnancies in 857 families were enrolled in our study; these foetuses were detected in the Prenatal Diagnosis Center in the First Affiliated Hospital of Zhengzhou University (Zhengzhou, China). This report may be considered the largest *DMD* gene mutation report from a single centre worldwide.

All probands were diagnosed with DMD/BMD based on the “Clinical practice guidelines for Duchenne muscular dystrophy” [7]. However, since the pathogenicity of the missense mutation of DMD cannot be determined at present, the proband diagnosed with the missense mutation was not included in the research scope. Then, we could provide guidance to these families with a DMD/BMD prenatal diagnosis.

All patients and their families signed informed consent. Additionally, this study was approved by the Ethics Committee of the First Affiliated Hospital of Zhengzhou University.

### Methods

#### Sample collection

EDTA-K2 tubes were used to collect 2 ml of peripheral venous blood from probands and pregnant females. A DNA purification kit (Tiangen Biotech Company, Beijing, China) was used to extract genomic DNA from leukocytes and buccal mucous cells in blood samples.

For women who were 10–14 weeks pregnant, chorionic villus sampling was performed under ultrasound guidance to obtain 10–15 mg of chorionic villi (amount 1–2; length 2–4 mm). For women who were 16–26 weeks pregnant, amniocentesis was carried out under ultrasound guidance to obtain 10–20 ml of amniotic fluid. A QIAamp DNA Mini Kit was used to extract foetal amniotic fluid/chorionic villus DNA (QIAGEN, Hilden, Germany).

The PowerPlex 16HS kit (produced by Promega company in Sweden) was used to eliminate maternal contamination. DNA samples were extracted according to the instructions of the DNA extraction kit (Omega Bio‑Tek, USA).

#### MLPA

MLPA was used to detect a large deletion or duplication of the *DMD* gene in probands, pregnant females and all foetuses. According to the manufacturer’s protocol, MLPA was performed with a SALSA MLPA Kit P034/P035 DMD/Becker (MRC-Holland, Amsterdam, The Netherlands). Amplification products were analysed on an ABI 3130 genetic analyser by capillary electrophoresis. The copy number was calculated according to the MLPA kit instructions, and the original data were analysed by Coffalyser.net and GeneMapper 4.0 software. Details of how to do this can be found in a previous article [8, 9].

#### NGS

NGS was used to detect small mutations of the *DMD* gene in probands and suspicious carriers with negative MLPA results. The Ion Ampliseq Designer (https://www.ampliseq.com) was used to design a *DMD* NGS gene panel to screen all 79 coding exons of the muscle-specific transcript NM_004006.2 and the 10 bp intron–exon junctions. An Ion PGM™ Sequencing 200 kit v2 was used on the sequencing platform for sequencing. Sequencing was performed using 500 flows. After sequencing using Ion PGM, using Ion Torrent Suite software v 4.0.2 to process the data. Details of how to do this can be found in a previous article [8].

#### Sanger sequencing verification

The loci with possible pathogenic mutations and loci with already known pathogenic mutations detected by NGS were verified by Sanger sequencing. An ABI G 3.1 sequencing kit was used to perform bidirectional sequencing of products on an ABI 3130xl DNA sequencer. The sequencing results were aligned with normal sequences by ABI Sequencing Analysis software v5.1.1.

#### STR linkage analysis

In the DMD/BMD prenatal diagnosis for families with a living proband, the combined results of linkage and mutation analyses can provide mutual verification. Linkage analysis performed on the family members and foetuses can be used to exclude no less than 10% maternal blood contamination. Eleven pairs of fluorescently labelled primers included six STR loci in the DMD gene and four STR loci on chromosome 21, namely, the repeat sequences 5′-(CA)_n_-3′,3′-MPIP, STR07A, STR44, STR45, STR49, STR50 and D21S1919, D21S236, D21S1914, D21S1255. The primer sequences are presented in Table 1. An ABI 3130xl genetic analyser and GeneMapper software v4.0 were used to analyse the data.

**Table 1:** The primer sequence of STR loci

Probe name	Length (in bp)	Forward/reverse primer (5′–3′)
5′-(CA)_n_-3′	206–228	tcttgatatatagggattatttgtgtttgttatac/attatgaaactataaggaataactcatttagc
DMDSTR07A	218–335	ttctggttttctggtctg/gaatcaatctctctgtcaag
STR-44	180–200	tccaacattggaaatcacatttcaa/tcatcacaaatagatgtttcacag
STR-45	152–178	gaggctataattctttaactttggc/ctctttccctctttattcatgttac
STR-49	223–260	cgtttaccagctcaaaatctcaac/catatgatacgattcgtgttttgc
STR-50	232–244	aaggttcctccagtaacagatttgg/tatgctacatagtatgtcctcagac
3′MP1P	65–81	atgatcagagtgagtaatcggttgg/atatcgatctagcagcaggaagctgaatg
D21S1919	168–198	agcggactgcatgaatcaca/gcgtctatcactctcatgttccta
D21S263	194–229	tacaagttaagggtgaaattggcta/gcgtcttcatcagcaagcgtcctctta
D21S1914	258–280	ccttctgtcacattatgaggaaaca/gcgtcttaaaggtatgattcactaaa
D21S1255	308–329	ctcttgattatgccacatagac/gcgtcttgctcgcagatgtttatta

#### Post-natal conformation

Venous blood samples of all newborns were collected to measure the level of serum creatine kinase and extract DNA for genetic diagnosis (MLPA or Sanger sequencing).

Aborted foetal tissues were collected to extract DNA for genetic diagnosis (MLPA or Sanger sequencing).

## Results

### Mutations in probands

Among all mutations in 857 families, large deletions, large duplications and small mutations accounted for 69.31% (594/857), 8.40% (72/857) and 22.29% (191/857), respectively. One patient was found to have a complex genomic rearrangement that involved a deletion of exons 45–50 and a duplication of exons 62–79. In addition, the number of deletions was far greater than the number of duplicates.

### Carrier statuses of pregnant females

A total of 857 pregnant females were enrolled in this study, including 575 (67.09%, 575/857) who exhibited the same *DMD* gene mutations as the proband, while the other 282 (32.91%, 282/857) did not carry the mutation (Table 2). Among them, peripheral blood of the mothers of all proband families was tested to demonstrate whether they had the same DMD gene mutation as the proband. The de novo rate was 32.91% (282/857) in our study.

**Table 2:** Genetic analysis of the DMD gene mutation

DMD gene mutation type	Mother were female carrier	Mother did not carry the mutation in peripheral blood	de novo mutation rate (%)
Exons deletion	351	243	40.91
Exons duplication	60	12	16.67
Small mutations	164	27	14.14
Total	575	282	32.91

### Results of prenatal diagnosis

Among 857 mothers with a childbirth history of DMD, 575 were carriers and 282 were not. Among all 931 foetuses (twins in one pregnancy were counted twice), 20.73% (193/931) were males expected to develop DMD, 16.33% (152/931) were female carriers, 37.59% (350/931) were males without *DMD* mutations and 25.35% (236/931) were females without *DMD* mutations. Moreover, 13 pregnant women (4.48%, 13/290) without DMD mutations who were at risk of having a foetus who was a carrier gave birth again, and 332 pregnant women (51.79%, 332/641) with DMD mutations gave birth again to normal foetuses. As DMD is an X-linked disease, it agrees with the law of Mendelian inheritance.

Five foetuses and the probands had the same *DMD* mutation, but their mothers did not carry mutations in peripheral blood. Hence, 0.83% (5/857) of the probands’ mothers had gonadal mosaicism. These results also indicated that 1.78% (5/282) of the probands’ mothers without mutations in peripheral blood had gonadal mosaicism. The mothers of the proband in these 5 families (1.51%, 5/660) were more likely to be gonad chimaeras.

A male foetus (*DMD*188) had a de novo deletion of *DMD* exons 45–50, which is completely different from the deletion of exons 3–29 previously identified in the proband and the mother who was a carrier. In addition, a male foetus whose mother did not have a family history had a de novo missense mutation of c.4529A > G (p. Lys1510Arg). According to the literature, this mutation is harmless.

**Fig. 1:**
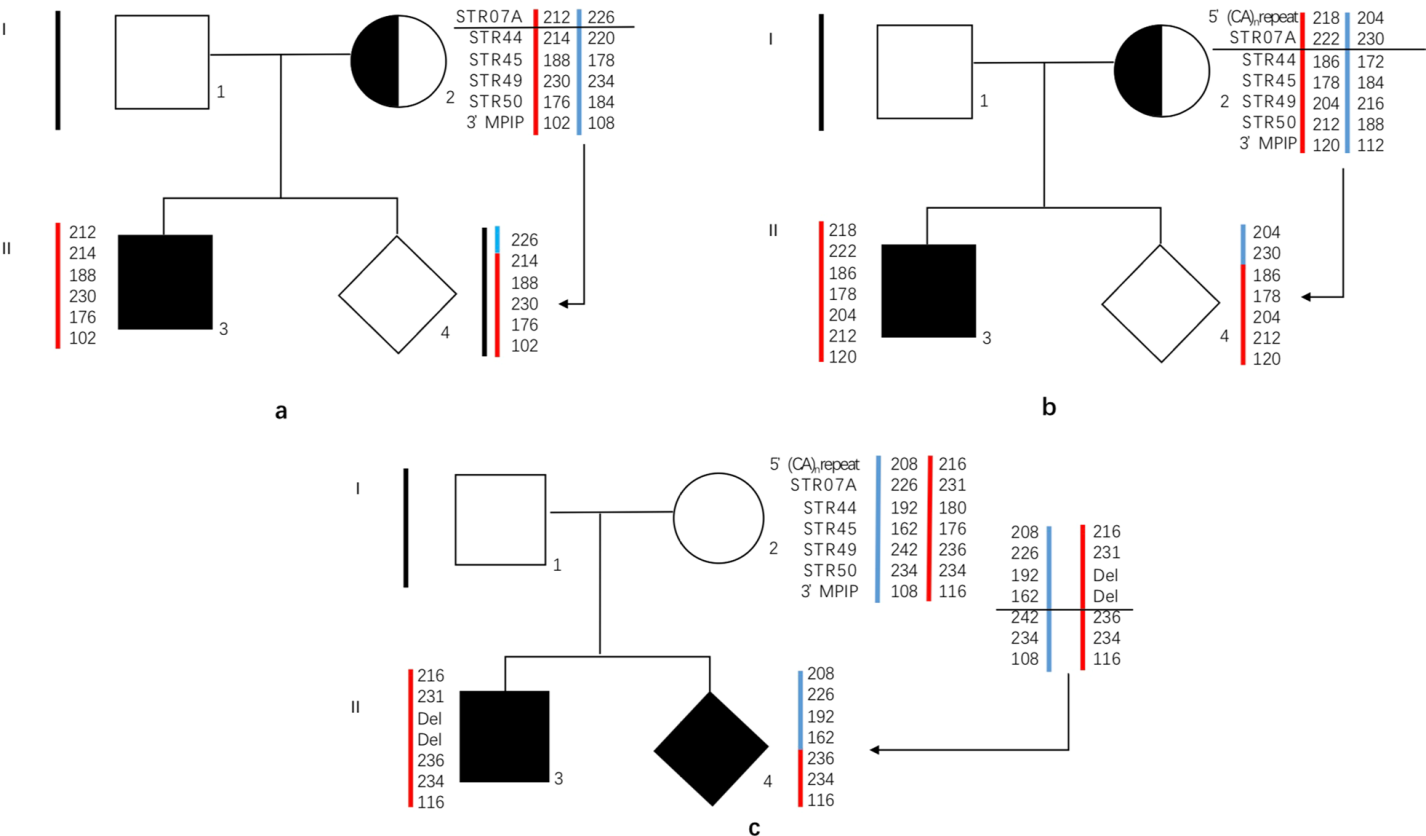
DMD gene recombination observed by STR linkage analysis. **a** The proband (II3) had the mutation c.8854C > T. The mother (I2) was a carrier. The same mutation was detected in the female fetus (II4) by Sanger sequencing. Linkage analysis showed that the fetus inherited the same high-risk haplotype as the affected proband at the following STR markers: STR44/STR45/STR49/STR50/3′MP1P, but the opposite allele at STR07 indicated that the fetus inherited a recombinant DMD gene. **b** The proband (II3) had the mutation c.1831G > T. The mother (I2) was a carrier. The same mutation cannot be detected in the male fetus (II4) by Sanger sequencing. Linkage analysis showed that the fetus inherited the same high-risk haplotype as the affected proband at the following STR markers: STR44/STR45/STR49/STR50/3′MP1P, but the opposite allele at 5′(CA)nrepeat and STR07 indicated that the fetus inherited a recombinant DMD gene. **c** The proband (II3) had the deletion of exon 45–47. The large deletion cannot be found in the mother’s (I2) peripheral blood. The male fetus had the deletion of exon47 detected by MLPA. Linkage analysis showed that the fetus inherited the same high-risk haplotype as the affected proband at the following STR markers: STR49/STR50/3′MP1P, but the opposite allele at 5′(CA)nrepeat/STR07/STR44/STR45 indicated that the fetus inherited a recombinant DMD gene

In addition, there was another female without *DMD* mutations in peripheral blood who gave birth to a proband with deletion of the *DMD* gene exons 45–47. MLPA analysis indicated that her foetus (*DMD*195) was a male with a deletion of exon 47 in the *DMD* gene. There are two possible explanations for this case. The first possibility is that the mother had gonadal mosaicism, and gene recombination occurred in the egg with the deletion of exons 45–47 and resulted in the de novo deletion of exon 47 in the foetus. The other possibility was that the two mutations of the proband and the foetus were de novo mutations, which were caused by two gene recombinations in normal eggs (Fig. 1c).

**Fig. 2:**
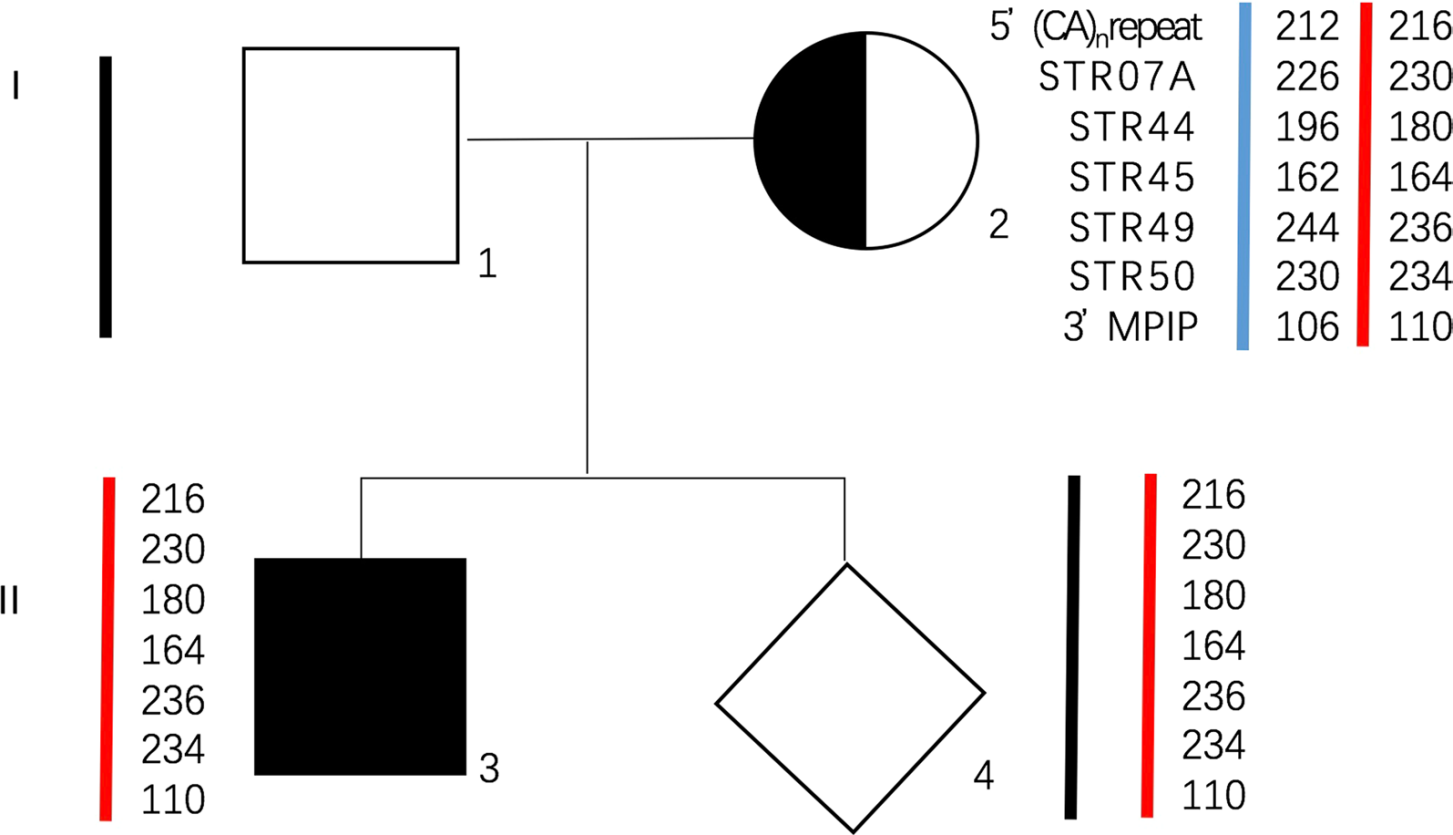
DMD gene recombination cannot be observed by STR linkage analysis. The proband (II3) had the the deletion of exon 46–48. The mother was a carrier. The same peak value of 5′(CA)nrepeat/STR07/STR44/STR45/STR49/STR50/3′MPIP indicated that the fetus inherited the same X chromosome from the mother. But the MLPA result of the female fetus (II4) was no deletion. We thought gene recombination occurred

STR linkage analysis was performed on the foetuses (DMD017, DMD195, DMD291), and the results indicated that gene recombination occurred (Fig. 1). The results of STR linkage analysis and MLPA were inconsistent in one foetus (DMD147). This event might have been caused by gene recombination, which cannot be observed in STR linkage analysis because of the limited STR locus (Fig. 2).

### Post-natal confirmation

The genetic diagnosis results of all newborns and aborted foetuses were consistent with the prenatal diagnosis results.

## Discussion

Many patients with a family history of DMD have visited our centre. If a proband was confirmed in a family, they would be worried about their next generation and strongly wish to determine the genetic causes to avoid the same disease. In the present study, data from patients with DMD were retrieved and analysed.

### Methods of *DMD* gene detection

MLPA is one of the most widely used methods [10], but it cannot be applied to the detection of very small mutations, while Sanger sequencing is expensive and time consuming in the detection of DMD gene point mutations. Compared with Sanger sequencing technology, NGS has the advantages of large flux, a short time, high accuracy and abundant information, which significantly shortens the detection cycle and reduces the detection cost [11, 12].

### The methods of DMD prenatal diagnosis

**Fig. 3:**
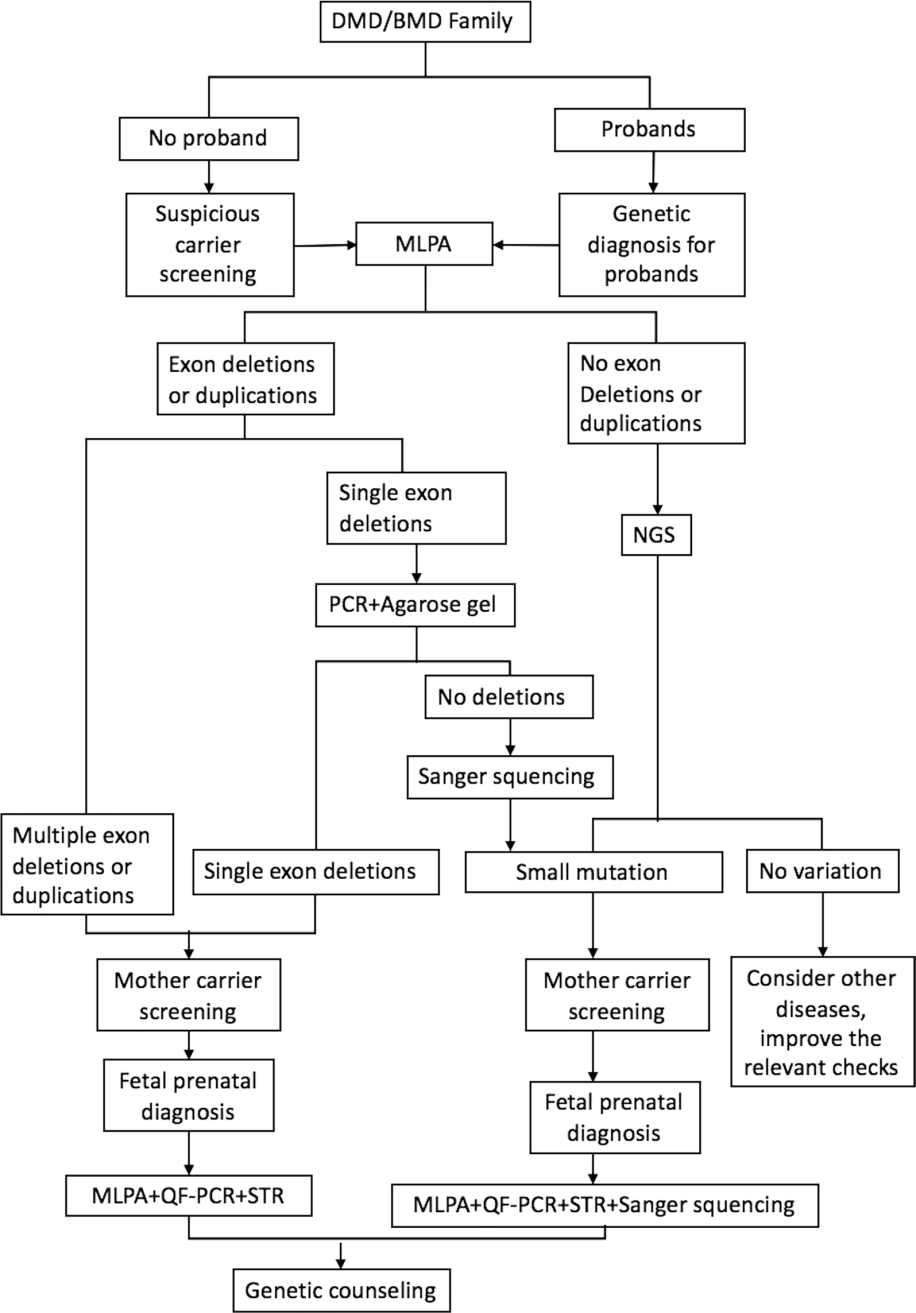
The procedure of genetic testing and prenatal diagnosis for DMD/BMD

DMD/BMD prenatal diagnosis can be classified into direct and indirect gene diagnoses. Methods of direct diagnosis, such as MLPA and Sanger sequencing, are the gold standard, but mutations in probands need to be defined to detect *DMD* gene mutations in the foetus [13]. NGS improves the efficiency of proband mutation detection [14, 15]. Indirect prenatal diagnosis predicts prenatal risks using STR linkage analysis, with the advantages of simplicity and quickness [16]. However, the risk of gene combinations cannot be ignored [17]. The different results between direct and indirect prenatal diagnoses in one foetus (DMD147) confirmed that separate STR linkage analysis had the risk of misdiagnosis in the DMD/BMD prenatal diagnosis. Therefore, the combination of direct diagnosis and indirect diagnosis is the current routine prenatal diagnostic strategy (Fig. 3).

In this research, MLPA was used to exclude de novo large deletions/duplications in the *DMD* gene for all foetuses. Based on the consideration of the high cost of NGS and the low percentage of small mutations among all de novo mutations, we did not use NGS to screen small de novo mutations for all DMD/BMD prenatal diagnoses. However, NGS was required by the families that had probands with small mutations.

### *DMD* gene mutation and genetic analyses

Among all 931 foetuses (twins in one pregnancy were counted twice), 20.73% (193/931) were males expected to develop dystrophinopathies, 16.33% (152/931) were female carriers, 37.59% (350/931) were males without *DMD* mutations and 25.35% (236/931) were females without *DMD* mutations. Moreover, 13 pregnant women (4.48%, 13/290) without DMD mutations at risk of having a foetus who was a carrier gave birth again. Thus, pregnant women without the *DMD* mutation have an extremely low chance of having another child at risk again. However, it is suggested that women who have given birth to a child with DMD need a prenatal diagnosis when she becomes pregnant again.

In our research, five mothers without *DMD* gene mutations in peripheral blood had a foetus with DMD, but their foetuses had the same *DMD* mutations as the proband. This result might be caused by gonadal mosaicism, but the possibility of de novo mutations cannot be ruled out. In addition, we cannot exclude the possibility of somatic mosaicism in these five mothers. In theory, MLPA and NGS can be used to exclude mosaicism. MLPA cannot distinguish the low percentage of mosaicism. However, no research has confirmed that NGS can be used to test somatic mosaicism. Thus, it was unfortunate that we could not exclude the possibility of somatic mosaicism in these five mothers. In summary, without considering the possibility of de novo mutations and somatic mosaicism, 1.78% of proband mothers without mutations in peripheral blood had gonadal mosaicism in our study. Our data were similar to another study that indicated a recurrence risk of 8.6% for non-carrier females due to germline mosaicism [18]. Thus, with the high de novo mutation rate, we suggest that a prenatal diagnosis is still necessary for families.

Our data indicated that 32.91% of DMD/BMD probands had de novo mutations, which was similar to previous reports [19, 20]. Among these de novo mutations, large deletions/duplications accounted for approximately 70%. These data and the discovery of a foetus with a de novo mutation (deletion of exons 45–50) (DMD188) that was different from the mutation in the probands and mother carriers (deletion of exons 3–29) illustrate the high de novo mutation rate of the *DMD* gene. Hence, de novo mutations in foetuses should be considered in prenatal diagnosis.

**Fig. 4:**
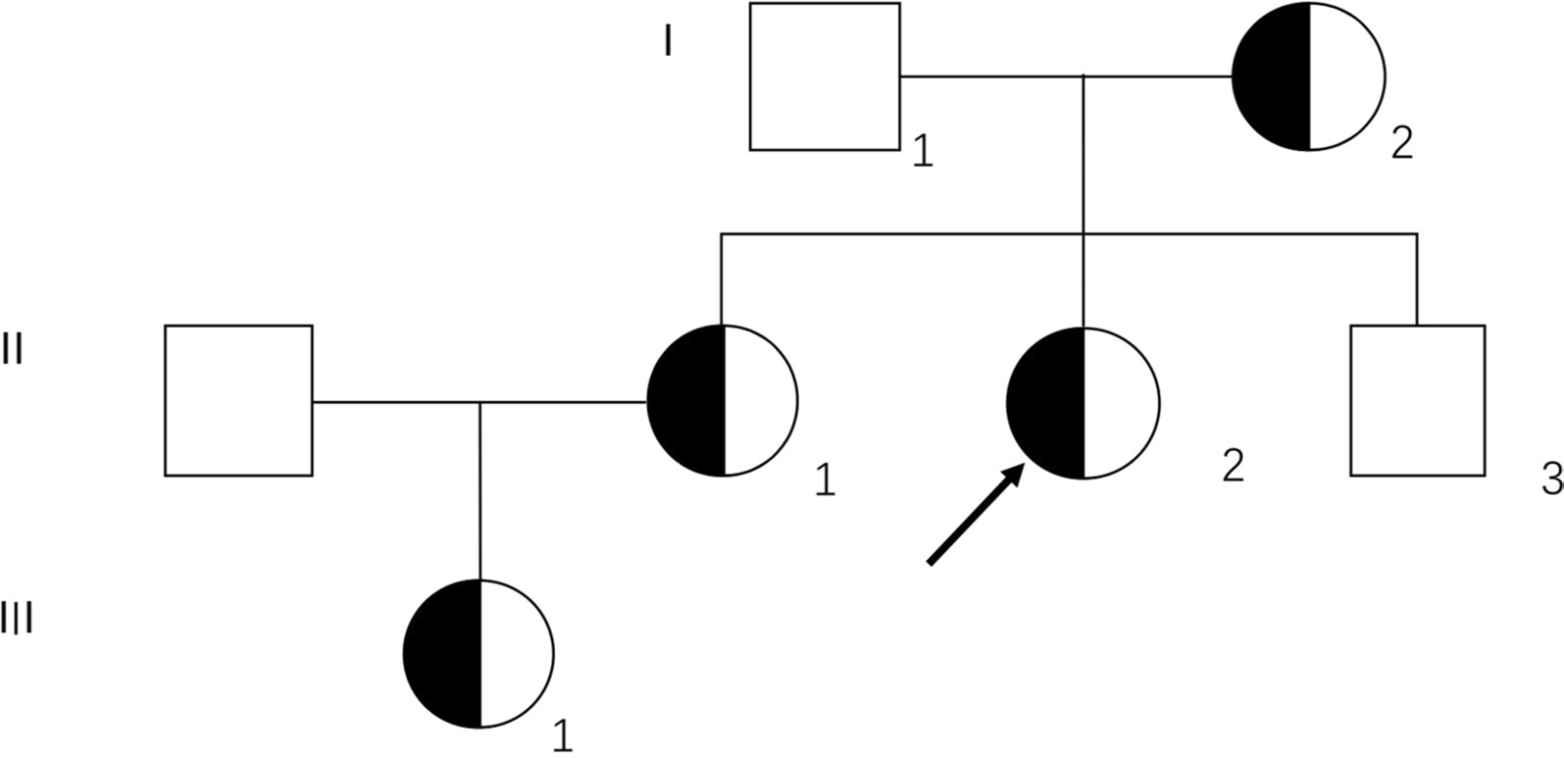
An atypical family with a female proband. The proband (II2) was 17-year-old female having the following symptoms: no ambulation, creatine kinase level of 5430 U/L (normal range 24–194 U/L), muscle biopsy indicating muscular dystrophy, immunohistochemical staining indicating dystrohpin completely absent. MLPA analysis showed that the proband carried heterozygous deletion of exons 8–21. Her mother (I2) and sister (II1) was also the mutation carrier but without any symptoms, and her sister’s creatine kinase level was 421U/L (normal range 24–194 U/L). The female fetus (III1) is a female carrier and the post-natal conformation illustrate that the female fetus was not affected

An atypical family (DMD303) with a DMD female proband (II2) was detected (Fig. 4). The 17-year-old female proband, her mother and her elder sister all carried the heterozygous deletion of exons 8–21. The female proband was clinically diagnosed with *DMD*, but her mother and elder sister had no symptoms. The prenatal diagnosis result indicated that the female foetus of her sister (III1) carried the deletion of the DMD gene exons 8–21. In genetic counselling, we told the above information and the low percentage of DMD female patients in the population, and only 3 female probands in our data chose to continue pregnancy and had a female infant after term delivery. The post-natal conformation of the female foetus was normal (creatine kinase level was 186 U/L). At present, the pathogenesis of symptomatic female DMD carriers is unclear, but some research has reported that approximately 20% of female DMD mutation carriers have varying degrees of clinical symptoms [21–23]. It is worth noting that prenatal diagnosis for families with DMD/BMD female patients is necessary and provides genetic counselling for such families.

### The expectation of a DMD prenatal diagnosis

In conclusion, the basic strategy in our study was to first use MLPA and NGS to identify DMD mutations in probands and then use MLPA and Sanger sequencing combined with STR linkage analysis to detect definite mutations for a DMD/BMD prenatal diagnosis. In this protocol, gene recombination, gonadal mosaicism and de novo mutations of the *DMD* gene were also taken into consideration. In general, prenatal diagnosis is currently limited to hereditary diseases. However, it is not practical for all pregnant women to have a prenatal diagnosis via amniotic fluid puncture. Although there are several treatments for DMD, the likelihood of a cure is extremely low.

Owing to the high de novo mutation rate of the *DMD* gene, the following questions are worth discussion: Should a prenatal diagnosis of DMD/BMD be limited to the defined *DMD* mutation locus in a family, or should it be extended to the full length of the *DMD* gene? Moreover, issues should be discussed regarding how to implement prenatal diagnosis and genetic counselling for DMD/BMD families with missense mutations and what principles should be met in the DMD/BMD prenatal diagnosis for families with female DMD/BMD patients.

In addition, the functional verification of missense mutations was lacking, and prenatal diagnosis was the only option for these families. However, since the pathogenicity of the missense mutation of DMD cannot be determined at present, the proband diagnosed with a missense mutation was not included in the research scope.

### The treatment expectation of DMD

In our study, 193 foetuses were diagnosed with DMD. In China, pregnant women have the option of having a voluntary termination of pregnancy. However, not all countries allow a voluntary termination of pregnancy. Therefore, many paediatric patients are still born ceaselessly. Hence, not only is a prenatal diagnosis crucial, but the treatment of patients should also be valued.

At present, there is no cure for DMD patients, and gene therapy is the only hope, which has attracted much attention worldwide. According to the structure of the DMD genome, DMD can be treated by splicing exons to restore the loss of the open reading frame (ORF), including the complete reading of nonsense mutations [24–27] (Ataluren (PTC124)) and skipping of exons [28–33] (Eteplirsen (Exondys 51) and Golodirsen (Vyondys 53). Ultimately, the DMD phenotype is converted to mild BMD. In addition, the percentages of patients treated by jumping exon 51 or the complete reading of nonsense mutations were 14.51% and 11.67%, respectively, which is almost consistent with the percentage of 15% [34] in Scoto M’s study and 16–17% in McDonald C M’s study [35].

## Conclusion

According to our study, it is suggested that the combination of MLPA, NGS, Sanger sequencing and STR linking is an efficient genetic diagnostic tool for DMD/BMD and provides a useful reference to further the diagnosis and treatment of DMD. Due to the high de novo rate of DMD, genetic counselling, pregnancy screening and prenatal diagnosis are necessary.

## Data Availability

The raw datasets generated during the current study are not publicly available because it is possible that individual privacy could be compromised. The Ion Ampliseq Designer (www.ampliseq.com).

## Abbreviations

DMD: Duchenne muscular dystrophy
BMD: Becker muscular dystrophy
MLPA: Multiplex ligation-dependent probe amplification technology
NGS: "Next-generation" sequencing
STR: Short tandem repeat
